# Rationale and Design of the Women’s Health And Daily Experiences Project: Protocol for an Ecological Momentary Assessment Study to Identify Real-Time Predictors of Midlife Women’s Physical Activity

**DOI:** 10.2196/19044

**Published:** 2020-10-15

**Authors:** Danielle Arigo, Megan M Brown, Kristen Pasko, Matthew Cole Ainsworth, Laura Travers, Adarsh Gupta, Danielle Symons Downs, Joshua M Smyth

**Affiliations:** 1 Department of Psychology Rowan University Glassboro, NJ United States; 2 Department of Family Medicine Rowan School of Osteopathic Medicine Stratford, NJ United States; 3 Departments of Kinesiology and Obstetrics & Gynecology Pennsylvania State University University Park, PA United States; 4 Departments of Biobehavioral Health and Medicine Pennsylvania State University University Park, PA United States

**Keywords:** women's health, midlife, cardiovascular risk, physical activity, ecological momentary assessment, mobile phone

## Abstract

**Background:**

Midlife women are at an elevated risk for cardiovascular disease (CVD) and associated mortality. Those who have additional risk conditions such as obesity or hypertension report specific barriers to engaging in cardioprotective behaviors such as physical activity (PA). Considerable effort has been devoted to understanding PA determinants and designing interventions for midlife women, although with suboptimal success, as increasing PA could meaningfully attenuate CVD risk. An updated approach to understanding PA among midlife women could improve upon existing resources by focusing on novel psychosocial influences on PA in this population (ie, body satisfaction, social interactions, social comparisons, mood state) and within-person relations between these influences and PA in the natural environment.

**Objective:**

The overarching goal of Project WHADE (Women’s Health And Daily Experiences) is to use an ecological momentary assessment (EMA) approach to capture ecologically valid relations between midlife women’s psychosocial experiences and PA as they engage in their normal daily activities. The primary aim of the study is to identify within-person psychosocial predictors of variability in PA (ie, experiences associated with higher vs lower PA for a given individual).

**Methods:**

Midlife women (aged 40-60 years) with one or more additional risk markers for CVD (eg, hypertension) will be recruited from primary care clinics and the general community (target n=100). Eligible women will complete an initial survey and a face-to-face baseline session before engaging in a 10-day EMA protocol. Psychosocial experiences will be assessed using a brief self-report via a smartphone 5 times per day, and PA will be assessed throughout waking hours using a research-grade monitor. Participants will return for a brief exit interview at the end of 10 days. Multilevel models that address the nested structure of EMA data will be used to evaluate the study aims.

**Results:**

Recruitment and enrollment are ongoing, and a total of 75 women have completed the protocol to date. Data collection is expected to be completed in Fall 2020.

**Conclusions:**

Project WHADE is designed to identify naturally occurring psychosocial experiences that predict short-term variability in midlife women’s PA. As such, the results of this study should advance the current understanding of PA among midlife women by providing further insight into within-person psychosocial influences on PA in this group. In the future, this information could help inform the design of interventions for this population.

**International Registered Report Identifier (IRRID):**

DERR1-10.2196/19044

## Introduction

This paper describes the rationale and design of Project WHADE (Women’s Health And Daily Experiences), an intensive, observational study designed to improve upon the current understanding of midlife women’s physical activity (PA) and inform PA interventions designed for this population. Midlife women (age 40-60 years [1]) currently account for the largest subset of US health care expenditures compared with other gender and age groups examined [2]. Much of their health care costs are associated with risk for cardiovascular disease (CVD), including conditions such as hypertension and type 2 diabetes [3]. During midlife, meaningful gender differences emerge with respect to CVD risk. For example, the onset of menopause confers a direct biological risk for women [4,5]. The risk of mortality from CVD events also increases more sharply for midlife women than men [6,7], in part because of gender differences in symptom presentation and concerns about seeking medical attention [8-11]. A focus on promoting cardioprotective behaviors among midlife women, particularly those who have additional risk factors for CVD (eg, hypertension), could help mitigate this gender disparity and reduce CVD mortality among women.

PA is a cardioprotective behavior known to attenuate CVD risk, as adequate levels of PA contribute to reducing CVD risk factors such as waist circumference and blood pressure [12] and improving metabolic functioning [13,14], particularly among women [15-17]. Despite these benefits, the gender difference in PA that is observed across the lifespan (with men engaging in more PA than women) widens during midlife [18,19]; many midlife women fall far short of the PA thresholds recommended for health [20,21] and are difficult to engage in PA programs [22]. Midlife women with additional CVD risk factors (eg, overweight or obesity, hypertension) tend to report low motivation for PA and a variety of barriers to PA engagement, such as difficulty with time amid professional responsibilities and family care (as primary caregivers) [23,24]. PA promotion work focused on addressing barriers such as lack of time and motivation (both among midlife women and in other populations) has generated meaningful insights but has not yet led to large PA increases that are sustained over time [25,26].

Increasing PA thus remains to be an important avenue for reducing midlife women’s risk for CVD, and using new approaches to understanding facilitators of and barriers to PA could meaningfully improve intervention efforts for this group. Project WHADE addresses this need in 2 ways. First, by focusing on midlife women’s psychosocial experiences that are indicated by both theory and evidence to serve as facilitators and barriers to their PA but are understudied in this group. These include *body satisfaction*, *perceptions of social interactions*, *social comparisons*, and *mood states*. Second, by assessing proposed predictors of PA at the *within-person* level (ie, fluctuations within the same person over short periods) rather than the between-person level (ie, global reports or averages across time). The typical approach to estimating PA engagement and its psychosocial correlates is for participants to wear a PA monitor for several days and then to average their PA across days of monitor wear to generate a between-person estimate. These averages are correlated with trait- or person-level psychosocial characteristics (eg, global level of motivation for PA or social support) to determine the type or types of individuals who do and do not achieve recommended levels of PA [27]. Interventions are then designed to address the person-level characteristics associated with low PA [28].

This approach assumes that the observed relations at the between-person level (which describes differences between people) correspond to the within-person level (which describes changes in the same person over a given time frame). In many cases, this assumption is incorrect. For example, although *individuals* who engage in PA more (vs less) often have lower resting heart rates, *when* a given individual engages in PA (vs sedentary behavior), their heart rate increases rather than decreases [29]. Similar discrepancies may exist concerning between- versus within-person relations between psychosocial experiences and PA; for instance, people who generally experience more (vs less) body satisfaction may also engage in more PA, although their PA may actually *decrease* at times when they experience more (vs less) body satisfaction. Failure to differentiate these processes and thus assuming the incorrect direction of a relation between a predictor and a health outcome such as PA could lead to inaccurate models of behavior change and ineffective interventions that are based on these models. In line with the body satisfaction example mentioned earlier, the between-person relation between body satisfaction and PA would suggest that improving an individual’s body image should improve their PA, whereas within-person relations suggest the opposite effect. Thus, clarifying between- versus within-person relations between midlife women’s psychosocial experiences and PA could improve both PA theory and interventions specific to this population [30] by more accurately specifying the levels and directions of these relations and the associated mechanisms for increasing PA. To this end, this study was designed to differentiate between- and within-person relations between midlife women’s PA and their naturalistic experiences of body satisfaction, perceptions of social interactions, social comparisons, and mood state.

### Facilitators and Barriers: Novel Psychosocial Influences on Midlife Women’s PA

Accumulating evidence shows the importance of specific psychosocial facilitators of and barriers to PA for midlife women that differ from those of men [31-33]. We first describe the psychosocial processes hypothesized to influence midlife women’s PA (which have received less attention in this group than motivation or time management) and then detail the within-person approach in Project WHADE to assessment and the associated rationale.

In line with multiple theoretical models of health behavior and behavior change processes [34,35] and their evidence bases as mechanisms of behavior change [36-40], critical psychosocial influences on PA include *body satisfaction*, *social interactions*, *social comparisons*, and *mood state*. For example, social cognitive theory is an overarching framework that emphasizes self-perceptions, emotions, and behavior in the context of input from the social environment, including perceived norms and modeling [34]. Social comparison theory expands on a particular aspect of social influence to explain how self-evaluations relative to others can motivate (or demotivate) health behaviors such as PA [35]. At present, however, relations between these experiences and PA rarely are captured using within-person methods, particularly among midlife women. Differentiating within- versus between-person relations between midlife women’s psychosocial experiences and PA would help to clarify whether and in what ways these experiences predict PA.

#### Body Satisfaction

Across the lifespan, women are more likely than men to use PA to manage weight or appearance [41,42]. Although physical changes (eg, weight gain) consequent to childbirth, aging, and menopause may make body satisfaction uniquely important for midlife women [43,44], most of the research on the relation between body satisfaction and PA has focused on younger women. This work has shown that PA may be most likely to occur in response to dissatisfaction with one’s weight or shape [45], as women may be motivated to engage in PA with the expectation that it will reduce weight or otherwise improve their appearance. However, among overweight women, embarrassment, shame, and fear of judgment from others often lead to PA avoidance rather than engagement [46,47]. Feeling satisfied with one’s body—a positive state that many interventions attempt to promote—also has been associated with future *decreases* in PA among young women [45]. Although body satisfaction traditionally has been conceptualized and measured as a stable difference between individuals, ample evidence indicates that body satisfaction fluctuates over short time frames and changes in response to context shifts [48] and deliberate intervention [49]. Ecological momentary assessment (EMA) methods have been used in several studies to assess within-person variability in body satisfaction among young women [50] but rarely have been employed among midlife women.

Thus, it appears that the relation between body satisfaction and PA is complex. Both high and low satisfaction could motivate PA, and the circumstances that determine the direction of the association are not yet clear, especially as they pertain to midlife women’s experiences in their natural environments. Furthermore, engaging in PA for appearance-related reasons is associated with worse physiological health [51] and a lower likelihood of maintaining PA in the long-term compared with engaging in PA for enjoyment or health [52,53]. As such, a better understanding of how this relation manifests in midlife women’s daily lives could eventually help to promote both healthy perceptions of one’s body and healthy engagement in PA.

#### Social Interactions

For many individuals, interactions with other people occur on and off throughout the day; some interactions are more meaningful than others, in that they prompt consequential thoughts, emotions, or behaviors, whereas other interactions have little detectable effect on these outcomes [54]. Emerging evidence suggests that perceptions of social interactions influence PA in that self-reports of individuals’ interpretations of the interaction or assessment of the positivity or negativity of the experience is associated with PA engagement [55]. Social influence may be exerted directly, via encouragement or discouragement of PA and other healthy behaviors, or may occur indirectly through creating norms (for or against PA) or limiting time or motivation for PA (eg, if negative interactions lead to stress or necessitate problem solving) [34,56]. For example, engaging in PA is a method for coping with negative experiences [41,42,57], including those that result from unpleasant social interactions, and women are more likely than men to use PA to cope with stress [58,59]. However, stronger (vs weaker) perceptions of positive social interactions are associated with adults’ future PA engagement [60], and within-person, older adults have been shown to engage in PA at times when they report positive (but not negative) social interactions [61]. Thus, limited evidence shows that positive social interactions are associated with PA engagement; the role of negative interactions is less clear, and the conditions under which either type of interaction facilitates versus hinders PA have yet to be pinpointed.

In addition, the role of social interactions in PA appears to differ between women and men. Among adolescents and young adults, women cite the influence of social interactions on their PA decisions more often than men [62], although women perceive less support for PA from their family and peers compared with men [63,64]. Research focused on young women also indicates that positive interactions tend to motivate PA, whereas negative interactions tend to increase stress and interfere with PA [62,65] and show that greater objectively assessed PA occurs on days with more (vs less) positive social interactions and less (vs more) negative interactions [55]. To date, however, this work has spanned several distinct characteristics of social interaction, including the presence versus absence of interactions, number of interactions in a given time frame, and the perceived positivity or negativity of a single interaction or set of interactions.

Moreover, although the social benefits of PA are also rated as more important among older versus younger women [66] and are consistently associated with PA during midlife [31,33,52], critical aspects of the relations between social interactions and PA remain to be unclear. Specifically, whether a within-person relations between social interactions and PA among midlife women is driven by positive or negative interactions (or both, independently or under different circumstances) and whether it is the *quantity* of either type of interaction or the perceived intensity (*quality*) of the interactions that drives these effects. These characteristics have been differentially associated with health and well-being outcomes in a range of populations [67-69]. As such, assessing their within-person relations with PA among midlife women would help to clarify the roles of distinct aspects of social interactions.

#### Social Comparisons

Social comparisons, or self-evaluations relative to others, represent an additional facet of social perception that may influence PA among midlife women. Comparisons with others are common in daily life and can be made across a range of domains (eg, appearance, wealth, work performance, health behaviors) and communication modes (eg, in person, via phone, via a social media platform) [70]. Social comparisons can have stronger effects on self-concept and behavior than comparisons with objective standards [71,72], and reported engagement in comparison shows both cross-sectional and prospective relations with health outcomes [35,73,74].

With respect to PA, social comparison may provide motivation via learning about discrepancies between one’s own PA engagement and that of others [61]. For example, comparisons with others viewed as doing better than the self (ie, upward comparisons) could indicate that improvement is possible and provide guidance and inspiration for achieving a similar goal [75]. Conversely, comparisons with others viewed as doing worse than the self (ie, downward comparisons) could boost self-efficacy for achievement and motivate behavior maintenance to avoid becoming like the worse-off other [76].

Experimental exposure to PA-based social comparison opportunities (eg, PA leaderboards, step competitions) shows positive group-level effects on subsequent PA and outperforms behavior change techniques such as enhancing social support when individuals are experimentally exposed to only 1 technique [77-79]. Such comparisons may be more influential for women than men; women report greater attention to [80] and interest in role models [46,47] and show stronger positive responses to same-gender role models [81] than men. Exposure to others engaged in PA also increases the likelihood that women will engage in PA, but this association is not observed among men [80]. Among young women, one’s tendency to make comparisons has been shown to change over 2 months, and this change mediates the effect of a brief intervention on unhealthy behaviors (eg, disordered eating and excessive exercise) [82]. Young women also show an ability to differentiate days and periods during which they recall making (vs not making) comparisons, and these distinctions are differentially associated with self-reported and objectively assessed PA [55,83]. Furthermore, shifts in the frequency of women’s PA-based social comparison opportunities from week to week are associated with changes in their PA over the same time frame [84].

However, not all individuals respond positively to social comparison opportunities [85], and the same individual may vary in their responses to comparisons over time [86]. In addition to the potential positive outcomes of upward comparisons described earlier, these comparisons can highlight the comparer’s worse-off status and generate demotivating frustration or hopelessness. Similarly, downward comparisons can signal that the comparer is already doing well (and thus, does not need to make much effort to maintain their status) or indicate that a negative future state is likely or inevitable (so efforts to prevent it seem futile) [87]. The contextual factors that determine positive versus negative responses to comparison are not yet clear, although some evidence suggests that the response may depend on the extent to which the comparer identifies with or contrasts themselves against the comparison target [75]. Specifically, focusing on similarities with an upward target (identification) and differences with a downward target (contrast) produces positive responses, whereas contrast with an upward target and identification with a downward target produce negative responses [88,89]. At present, evidence consistent with this model of comparison is restricted to self-reported affect and motivation for health behaviors, and its application to objectively assessed health behavior (such as PA) has not been evaluated.

Taken together, existing evidence indicates that social comparisons may have a meaningful influence on PA and that this influence may be stronger for women than men. However, relations between comparisons and PA have rarely been examined among midlife women, especially at the within-person level. Greater attention to social comparison in this context could help to clarify the extent of its influence on PA in an at-risk group and provide needed insight into contextual factors that may explain previous equivocal findings (eg, identification and contrast processes).

#### Mood State

Mood state describes an individual’s immediate emotional experience or a combination of emotional experiences (eg, happiness, sadness, anger). These may remain stable over periods of weeks or months or fluctuate in response to daily or momentary changes in context [90,91]. Mood state is recognized as a primary and proximal determinant of behavior [92] that can be modified either directly (eg, relaxation exercises to reduce feelings of anxiety or stress) or indirectly (eg, by adjusting antecedent thoughts that give rise to a range of emotional experiences) [92].

Several different mood states have been shown to predict PA over a range of time frames [93]. For example, experiencing positive mood states, such as happiness or contentment, is generally associated with future PA engagement (vs sedentary behavior) [94-96], although greater variability (vs stability) in positive mood states has shown negative associations with future PA [97]. Evidence with respect to relations between other mood states and PA is more equivocal. The experience of stress often predicts lower PA, such that increases in perceived stress are associated with future decreases in PA [57]. However, a number of studies also document the opposite relation (ie, increases in stress associated with future increases in PA), suggesting that some PA engagement may be used as a stress management technique [57]. Among older women (aged 65 years and older), PA is cited as an antidote to anxiety about the aging process, reflecting a similar emotion regulation function of PA [98]. Although considerable research has shown that PA engagement is negatively associated with concurrent and future negative affect [99-101], there has been less empirical investigation of whether negative affect (or positive affect) prospectively predicts PA [94].

Research specific to midlife women confirms that PA engagement is inversely associated with negative affect in this population [102-104]. However, as examinations of prospective relations between mood states and future PA engagement among midlife women are scarce, it is not yet clear which mood states (and contexts for these states) are associated with women’s PA during midlife. For example, midlife women may be more likely to engage in PA when they experience positive (vs negative) emotions, or they may use PA to manage negative emotions (eg, stress) or physical symptoms (eg, menopause), or both, under different circumstances [105]. A better understanding of relations between mood state and subsequent PA for midlife women and contextual moderators of this relation (eg, certain types of social interactions) would help to clarify emotional predictors of PA in an at-risk group.

### Assessing PA Influences Among Midlife Women Using Within-Person Methods

Recent evidence shows that when assessed repeatedly over hours and days, both PA and each of the psychosocial experiences described above (body satisfaction, social interactions, social comparisons, and mood state) can vary considerably within the same person [50,70,106,107], and relations at the between-person level may not translate to the within-person level. For example, a stronger (vs weaker) between-person tendency to make upward comparisons is associated with greater engagement in PA [108], although experiencing an upward comparison may be associated with less PA in the short term (if an individual engages in upward contrast) [75,109]. As such, additional work to clarify the nature and extent of relations between psychosocial experiences and PA will be most accurate and informative if it captures variability at both levels and differentiates within- from between-person relations. This requires repeated assessments of the same individual over multiple time points, gathering data as relevant experiences that occur in daily life. EMA is one such method that typically prompts self-reports multiple times per day. This limits the window for recall and the need for the respondent to aggregate across experiences, as required by global (person-level) self-report [54].

In an EMA design, self-reports can be prompted by signals to handheld devices such as mobile phones. The timing of responses can be verified by this technology, and item responses can be examined for temporal associations with one another or with other ambulatory assessments (eg, behavior in the real world). Examining within-person associations between EMA survey responses (eg, capturing psychosocial experiences) and objectively assessed PA in the natural environment is an underused approach to understanding PA variability and its influences in real time [110]. Although previous work has established the feasibility of using EMA with midlife women [111-113], to our knowledge, an EMA approach to understanding psychosocial predictors of PA variability has not yet been used among women in this life stage with elevated disease risk [114].

The primary aim of this study is to examine within-person relations between midlife women’s psychosocial experiences (ie, body satisfaction, social comparison, social interactions, and mood state) and their PA to identify moment-level experiences associated with higher versus lower PA for this population. Secondary aims are to determine the timing and duration of these effects and to examine person-level moderators of within-person relations. Specific research questions to be addressed in this study include the following:

1. To what extent do body satisfaction, perceptions of social interactions, social comparisons, and mood state vary between- versus within-person among midlife women with elevated CVD risk?
2. To what extent does PA vary between- versus within-person among midlife women with elevated CVD risk?
3. Are moment-level differences in body satisfaction, perceptions of social interactions, social comparisons, and mood state related to differences in PA for these women (within-person)?
4. Do person-level characteristics such as age, BMI, or menopause status moderate within-person relations between psychosocial experiences and PA for these women?

### Objective

The purpose of this paper is to present the protocol for an EMA study designed to capture within-person relations between psychosocial experiences (ie, body satisfaction, social interactions, social comparisons, mood state) and PA among midlife women with elevated cardiovascular risk.

## Methods

### Project Overview

Project WHADE is a study of relations between midlife women’s psychosocial experiences and PA in everyday life. Midlife women (aged 40-60 years) with one or more CVD risk markers complete a 10-day EMA procedure, whereby they self-report on their recent body satisfaction, social interactions, social comparisons, and mood at 5 semirandom times per day. For the same 10 days, they also wear a waistband accelerometer to capture their PA during waking hours. All study activities were approved by the Rowan University and Rowan School of Osteopathic Medicine institutional review boards (approval Pro2018002377).

### Target Population and Eligibility

Women are eligible if they are aged 40 to 60 years (inclusive) and have one or more additional cardiovascular risk factors. These include smoking (current or quit within the past 3 months) or diagnoses of prediabetes, type 2 diabetes, hypertension, prehypertension, hyperlipidemia, hypercholesterolemia, or metabolic syndrome. Additional inclusion criteria were English language proficiency, no medical contraindications to PA, not currently pregnant, not currently engaged in a formal weight loss program, no comorbid medical conditions or psychiatric symptoms that would impede participation (eg, injury, active psychosis), and the ability to complete momentary electronic surveys via a personal mobile device (eg, smartphone or tablet). Individuals are excluded from participation if they do not meet the above criteria or if they state an intention to move away from the geographic area during the study period. Eligibility was not limited by current level of PA engagement, as existing evidence and pilot work suggested that engagement would range from low to moderate for the target population [115,116].

### Sample Size and Recruitment

The recruitment goal for this study is 100 participants and still ongoing. The sample size was calculated for cross-level multilevel models (described below) following estimates from simulations by Hox [117,118]. Using conservative estimates of compliance from previous studies and our pilot data (ie, 80% of surveys completed in the correct time window [111,113]), a sample size of 100 participants (level 3) with 10 days of observation (level 2) 5 times per day (level 1) would generate a minimum of 4000 completed surveys (maximum 5000). This number of observations exceeds the thresholds identified for detecting between-person (level 3) moderators of time-sensitive relations (level 1).

This study uses 2 primary recruitment strategies: web or print advertisements and direct referrals from providers in family medicine clinics. Web advertisements include a study-specific webpage, email announcements for employees and students at the supporting institution, social media posts (eg, Craigslist, Twitter, Facebook), and advertisements on local news websites. Print advertisements (ie, flyers, postcards) are posted in public locations such as libraries and community centers and appear in local newspapers. All advertisements provide phone and email contact information for the research team and offer interested individuals the option to complete an initial survey as their first step (see below).

Direct referrals take place on-site in family medicine clinics run by Rowan School of Osteopathic Medicine. Study collaborators identify patients with upcoming appointments who meet the eligibility criteria (based on chart review) and provide them with study information following their medical visit. Study staff are available on certain days of the week to provide additional information to interested individuals and to schedule telephone screenings.

### Telephone Screening

Potential participants are asked to complete a 10-min telephone screening with trained research staff, who verify eligibility, explain study procedures, and answer questions. Those who remain interested in participating are scheduled for a face-to-face setup appointment (baseline) and are directed to complete an initial electronic survey before their appointment. Women who take the initial survey as their first expression of interest receive a follow-up call and/or email from study staff to complete or schedule the screening call, respectively.

### Initial Survey

Eligible women who engage in telephone screening and schedule an initial appointment are sent a Qualtrics survey link via email. This survey assesses demographics, contact information, recent psychosocial and physical experiences (eg, symptoms of depression and anxiety, health-related quality of life), global social perceptions (eg, social support, social comparison tendencies), and previous experience with PA promotion programs (self-guided and professionally supported). See Table 1 for additional description of the measures included. Study staff monitors for completion of this survey before the scheduled baseline appointment and prompts potential participants via phone or email if it is not completed by the morning of the appointment. As noted, the survey is also available as a first point of contact. For those who complete the survey first, study staff use the contact information entered into the survey to conduct or schedule the telephone screening.

**Table 1:** Baseline measures.

Construct	Description	Measure (reference)
Demographics	Age, self-reported height and weight, income, education, marital status, ethnicity, race, medical conditions, menopause status	Developed for this study
Social media behavior	Frequency of engagement with various social media platforms Example: How often to you use each of the following? (Facebook, Twitter, etc.) – Less than one day per week (1) to More than once per day (7)	Developed for this study
Health-related quality of life	Physical and mental quality of life over the past four weeks	SF-36^a^ Health Survey [119]
Activity barriers	Factors that are perceived to get in the way of PA^b^ engagement	Barriers to Being Active Scale [120]
Exercise motivation	Expected outcomes of PA	Outcome Expectancies for Exercise Scale [121]
Lapses in exercise	Successful and unsuccessful attempts to increase exercise in the past year Example: In the past year, how many times have you started a formal exercise program, such as joining an ongoing group at a gym? (Numeric entry)	Developed for this study
Anxiety	Symptoms of anxiety over the past month	Beck Anxiety Inventory [122]
Body image	Perceived influence of body image on quality of life	Body Image Quality of Life Inventory [48]
Depression	Symptoms of depression over the past four weeks	Center for Epidemiologic Studies Depression Scale [123]
Perceived stress	Perceived intensity of stress over the past month	Perceived Stress Scale [124]
Sleep quality	Subjective sleep quality and intensity of sleep disturbances over the past month	Pittsburgh Sleep Quality Index [125]
Problem orientation	Attitudes related to problem solving approach and abilities	Problem Orientation Questionnaire [126]
Social comparison orientation	Tendency to make social comparisons and value information from them	Iowa-Netherlands Social Comparison Orientation Measure [127]
Social support	Perceived support from family and friends	Social Support Appraisals Scale [128]

^a^SF-36: 36-Item Short-Form Health Survey.

^b^PA: physical activity.

### Baseline Appointment

Women who complete the initial survey attend an on-site, 1-hour individual session at a research center with a trained staff member. During the visit, the staff member obtains written informed consent, measures the participant’s height and weight, and reviews study procedures. This includes wear and care of accelerometer and a detailed explanation of survey items. Instructions indicate that participants should complete EMA surveys within 1 hour of receiving them. This window for completing a survey was based on feasibility protocols from similar populations [111] and on pilot work with the population of interest to maximize survey completion within the context of participants’ work and personal commitments.

**Figure 1:**
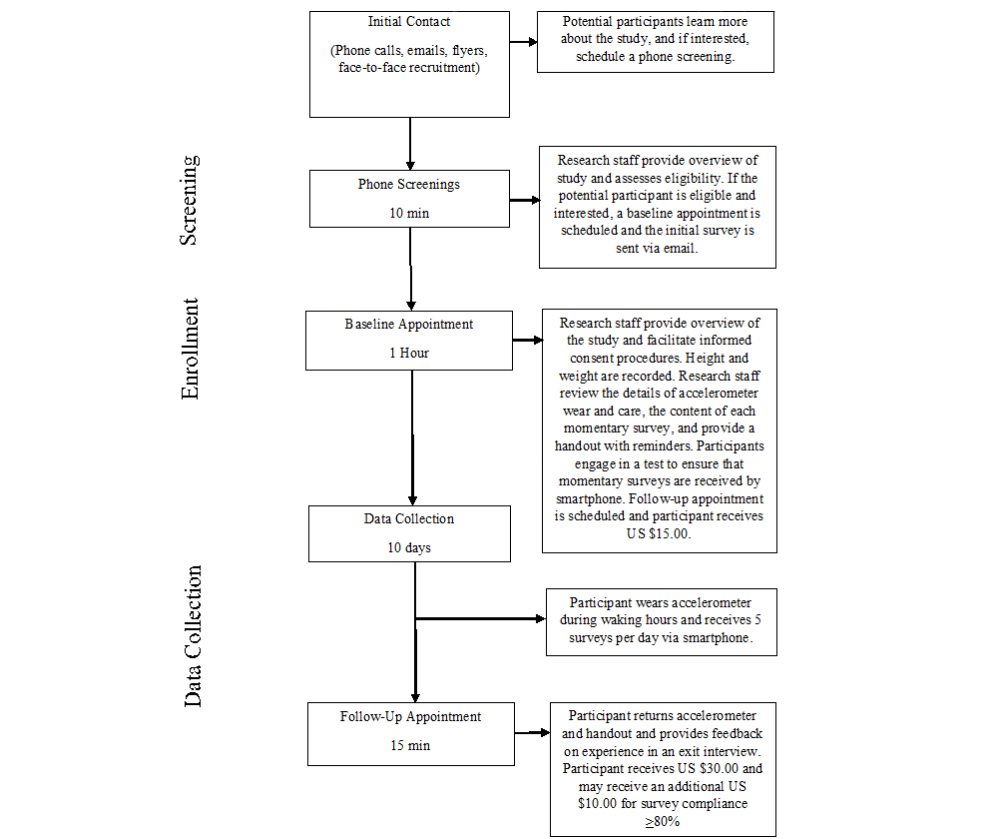
Screening, enrollment, and data collection procedures. EMA: ecological momentary assessment.

Participants are also given a folder with study materials to take home (eg, reminders from the baseline discussion, frequently asked questions about the accelerometer, an accelerometer wear log). The 10 days of EMA data collection in the participant’s natural environment are intended to begin the day after the participant’s baseline appointment. At the end of the baseline visit, participants are scheduled for a 15-min follow-up appointment to take place after the last day of the EMA data collection. See Figure 1 for a timeline of enrollment and data collection procedures.

### EMA Survey Schedule, Distribution, and Monitoring

The design of this study uses a signal-contingent approach, such that participants complete surveys in response to a signal (ie, text message). EMA surveys are scheduled and distributed via Qualtrics. Before study initiation, study staff generated survey schedules with semirandom timings anchored to 1 of 3 sets of specified times. These times were selected based on pilot work with the population of interest, which informed the expected frequency of events such as social comparisons, and were intended to maximize coverage of waking hours while minimizing reporting windows (and limiting retrospective recall) [129]. The sets of times were based on standard sleep or wake times for a 9:00 AM to 5:00 PM work schedule (8:30 AM, 12:15 PM, 3:45 PM, 6:30 PM, and 9:45 PM), an early-rising schedule (6:30 AM, 9:15 AM, noon, 3:30 PM, and 6:45 PM), and a late-rising schedule (11:00 AM, 2:35 PM, 5:45 PM, 8:45 PM, and 11:15 PM). The staff created 3 versions of each schedule and use a random number generator to assign 1 of the 3 survey schedules that match a participant’s sleep or wake schedule. Survey schedules do not differ between weekdays and weekends.

Survey distribution via text message with embedded web links is programmed immediately after a participant’s baseline appointment, and survey completion is monitored over the next 10 days. Research staff manually identify surveys outside of the 1-hour time window for reporting and surveys that repeat within the same 3-hour window (duplicates) for exclusion from statistical analyses.

### Follow-Up Appointment

After completing the 10-day accelerometer and survey protocol, participants return to the research center for their scheduled follow-up visit. Participants are asked to bring their accelerometer and a record of wear. A staff member conducts a brief exit interview to assess any difficulties, answer questions, and gather information about the participant’s preferences for future PA interventions. Participants who express interest in receiving mental and/or physical health resources are provided with materials sent via email. Those who indicate that they would like to view their PA engagement data are provided with a copy of their daily summaries. Summaries include daily totals for steps, sedentary time, and time spent in light, moderate, and vigorous activities.

### Compensation

Participants receive up to US $55 as compensation. They receive US $15 for attending the baseline appointment and US $30 when they return for follow-up. Those who return their accelerometers and complete more than 80% of EMA surveys receive an additional US $10 bonus at follow-up.

#### EMA Survey: Self-Rated Measures

EMA items were initially based on those used in previous intensive longitudinal assessment studies and were pretested for revision with the target population in 2 ways. Initial items were generated using previous work as a guide and were distributed in a 7-day pilot EMA study with women who met the criteria for this study (June 2017-May 2018; n=13). These items were revised based on participant feedback, and modified items were discussed in individual face-to-face interviews with a different group of women who met the criteria for this study (October 2018-December 2018; n=10). Interview feedback informed further item refinement, and the items used in the EMA surveys are presented in Textbox 1. EMA surveys were designed to take no more than 5 min to complete. The second, third, and fourth surveys each day included 29 items each; additional items in the first and last surveys of the day brought the totals for these surveys to 30 and 32, respectively.

**Ecological momentary assessment survey construct and items.**

Sleep quality (first survey of the day only):

- How would you describe your sleep last night?
  Excellent
  Good
  Average
  Poor
  Terrible

Mood state:

- How much have you experienced the following emotions: angry or frustrated, sad, happy or excited, content, anxious or stressed?
  Not at all
  Somewhat
  Very much

Pain:

- Have you had any physical pain in the last three hours?
  Yes
  No
- If yes, which areas of your body? List all that apply.
- If you have had pain, please rate the overall severity of your pain:
  Mild
  Moderate
  Severe
  Very severe

Positive social interactions:

- Did you have positive or pleasant social experiences with any of the following today?
  Friends
  Coworkers
  Acquaintances
  Family
  Strangers
  Other
- Total number of times you had positive or pleasant social experiences in the last three hours
- Overall, how positive or pleasant were your social experiences in the last three hours?
  Not at all
  Moderately
  Very

Negative social interactions:

- Did you have negative or unpleasant social experiences with any of the following today?
  Friends
  Coworkers
  Acquaintances
  Family
  Strangers
  Other
- Total number of times you had negative or unpleasant social experiences in the last three hours
- Overall, how negative or unpleasant were your social experiences in the last three hours?
  Not at all
  Moderately
  Very

Social comparisons:

- In the last three hours, did you think about or evaluate yourself or your behavior in comparison to someone else (or someone else in comparison to yourself)?
  Yes
  No
- How many times did you compare yourself to someone else in the last three hours?
- Did you communicate with the person you compared to?
  Yes. I talked to them in person, or on the telephone, or online (eg, Facebook message)
  No. I saw, heard about, read about, or thought about them but did not communicate
  Both
- What aspects of yourself did you compare? Select all that apply.
  Appearances
  Health habits
  Status
  Emotions
  Personality
  Abilities
  Other
- Below are some interpretations of the comparisons you may have made. Please indicate how many of each type you made since the last time you responded. Comparisons to people who
  Seem to be doing better than I am
  Seem to be doing about the same as I am
  Seem to be doing worse than I am

Most recent comparison:

- Now consider only your most recent comparison. Did you communicate with the person you compared to?
  Yes. I talked to them in person, or on the telephone, or online (eg, Facebook message)
  No. I saw, heard about, read about, or thought about them but did not communicate
  Both
- What aspects of yourself did you compare? Select all that apply.
  Appearances
  Health habits
  Status
  Emotions
  Personality
  Abilities
  Other
- As you compared yourself, how much did you focus on each of the following: how similar I am to the person I compared to; how different I am to the person I compared to?
  Not at all
  Somewhat
  Very much
- After the comparison, how much did you feel each of the following: inspired, encouraged, or hopeful about my own situation; anxious, frustrated, or discouraged about my own situation?
  Not at all
  Somewhat
  Very much

Motivation for physical activity (PA):

- How motivated are you to be physically active in the next few hours?
  Not at all
  A little bit
  Somewhat
  Very

PA intentions:

- Do you have plans to do cardiovascular exercise in the next few hours (such as going for a brisk walk or doing a strength DVD routine)?
  No, no plans to exercise
  Yes
- If yes, how many planned minutes?
- If yes, what kind of exercise?

Overall rating of the day (last survey of the day only):

- Was today a typical day for you, with respect to physical activity or exercise?
  Not at all. A lot less active than usual
  Mostly. A little less active than usual
  It was a typical day
  Mostly. A little more active than usual
  Not at all. A lot more active than usual
- Was today a typical day for you, with respect to eating?
  Not at all. A lot worse than usual
  Mostly. A little worse than usual
  It was a typical day
  Mostly. A little better than usual
  Not at all. A lot better than usual
  Number of meals eaten today
  Number of snacks eaten today

#### Body Satisfaction

Current body satisfaction is assessed with 1 item (“How would you describe your body satisfaction right now?”). Responses are rated on a 4-point scale ranging from 1 (*very dissatisfied with my body*) to 4 (*very satisfied with my body*) [50,130].

#### Social Interactions

Perceptions of social interactions since waking up (first survey of the day) or in the last 3 hours (all subsequent surveys of the day) are assessed with 6 items; 3 items focus on *positive or pleasant* interactions, and 3 on *negative or unpleasant* interactions. For each type, participants are asked to report the category or categories of others they interacted with (eg, family, friends, coworkers, strangers), the number of individual interactions they recall in the respective time frame, and the overall intensity of the interactions in that time frame (“How positive/pleasant were these experiences?”). The latter is rated on a scale of 1 (*not at all*) to 3 (*very*). Instructions specify that the number of interactions refers to the number of events, rather than the number of people, such that an interaction with a group counts as a single instance [55,61,131].

#### Social Comparison

Occurrence, type, and response to social comparison are assessed with 10 items. Participants are asked to report how many times they made social comparisons since they woke up (first survey of the day) or in the last 3 hours (all subsequent surveys of the day). Instructions specify that comparison includes any instance of evaluating an aspect of the self or one’s own behavior relative to that of others and that some comparisons might prompt emotional responses, whereas others might not. Both types should be counted in participants’ responses. Those who report one or more comparisons are asked what aspect or aspects of the self they compared (eg, appearance, health habits, abilities, etc) and the direction or directions of their comparisons (if they perceived the target to be upward, lateral [same as the self], or downward). Participants are also asked to provide these details about their most recent comparison before completing the survey, with additional items related to identification or contrast processes and affective response to the comparison (Textbox 1) [70,132,133].

#### Mood State

Recent mood state is assessed with 5 items, referring to how much participants have experienced each emotion since they woke up (first survey of the day) or in the last three hours (all subsequent surveys of the day). Participants are asked to report on a three-point scale ranging from 1 (*not at all*) to 3 (*very much*) for the following mood states: angry, happy or excited, stressed or anxious, sad, and content [134,135].

#### PA Motivation and Intentions

These experiences are assessed using 4 items. First, participants are asked to report how motivated they are to be physically active within the next few hours (surveys 1-4 of the day) or on the following day (survey 5 of the day). Second, participants are asked whether they have intentions of doing cardiovascular exercise in the relevant time frame; if they answer “yes,” they are asked to record the number of minutes they plan to exercise and the type of exercise they plan to do (eg, walking, taking an exercise class) [89,136-138].

#### Additional Experiences Assessed

EMA surveys also query for additional experiences that may affect engagement in PA and end-of-day reports on PA and other health behaviors. These include sleep quality (first survey of the day), pain (occurrence, location or locations, and intensity; all surveys), perception of whether the day was typical with respect to PA and eating behavior (last survey of the day), and the number of meals and snacks consumed that day (last survey of the day) [139-141].

### Activity Monitor

PA is assessed using the ActiGraph GT3X triaxial accelerometer (ActiGraph Corporation). Participants are instructed to wear the device aligned with their dominant hip during waking hours for 10 days following their baseline visit. They are asked to keep the device near their beds to limit forgetting to put it on upon waking, and to remove it for activities such as showering and swimming. Participants are also asked to complete a paper log for any time they remove the device for longer than 15 min during their waking hours, which is provided in the folder of study materials to bring home with them. PA parameters, including minutes of sedentary, light, moderate, and vigorous activity, are calculated using the ActiPro package for R. Moderate and vigorous minutes will be combined to estimate moderate-to-vigorous intensity physical activity. Time frames of interest include the concurrent reporting window (ie, 3 hours before each survey) and 30, 60, and 120 min after each survey to clarify the timing and duration of any observed effects [142].

### Data Analysis Plan

Multilevel models will be used to address the nested structure of self-report and accelerometer data: momentary assessments (level 1) within days (level 2) within individuals (level 3). Missing data patterns will be evaluated, and all models will employ maximum likelihood estimation techniques, which include all available cases and are robust to missing data (such as missed surveys or missed items within surveys). Initial empty models will evaluate the proportion of variance accounted for at each level for each PA parameter using the intraclass correlation coefficient. Within-person relations between psychosocial determinants and PA will be tested (in separate models for each PA parameter) by controlling for person-level covariates and the stable, person-level association between the predictor of interest and PA. These models allow for the identification of moment-level differences from an individual’s average that are associated with higher- or lower-than-average PA. Age, BMI, menopausal status, and the number of CVD risk factors will be considered as covariates and may be examined as person-level moderators of within-person relations.

## Results

To date, 172 women have expressed interest in participating via email, phone, family medicine clinics, or the initial survey. Of these women, 101 were contacted for screening and scheduled a baseline appointment; 76 women attended the appointment, and 75 of then completed the full EMA protocol and returned for their follow-up appointment (99% retention). Most of the enrolled participants identified as white (56/75, 75%) and married (44/75, 59%); 23% (17/75) reported household incomes of less than US $50,000, and 23/75 (31%) did not finish a bachelor’s degree program or received an associate’s or technical degree. The largest subsets of participants qualify as obese (52/75, 69%), postmenopausal (29/75, 39%), and report a previous diagnosis of high cholesterol (39/75, 52%). Recruitment and enrollment will continue until the target sample size of 100 is reached (expected in Fall 2020).

## Discussion

Project WHADE is designed to assess 4 psychosocial experiences that are hypothesized to predict midlife women’s PA: body satisfaction, social interactions, social comparison, and mood state. This paper describes our approach to understanding within-person relations between these experiences and PA and highlights existing challenges to be considered in future work.

### Methodological Challenges and Decisions

Given that EMA for capturing psychosocial influences on PA remains to be somewhat novel compared with other methods (eg, retrospective self-report, group-based experimental designs), there is limited evidence to inform key methodological decisions such as item wording for the constructs of interest, participant instructions, or optimal survey frequency and timing. As noted, we based the wording of our EMA items on those from existing, relevant studies and subjected them to 2 rounds of pilot testing with the population of interest. This also allowed us to gather feedback and estimates of survey and accelerometer compliance with the timing selected (ie, surveys 5 times per day and accelerometer wear for 10 days), which was intended to cover a representative subset of participants’ typical experiences while maximizing power and minimizing recording burden [143]. In addition, we trained study staff to provide participants with scripted, detailed explanations of each item at baseline visits to limit individual differences in how participants understand each psychosocial construct and item; it is not clear how widespread such procedures are or how effective they might be for reducing reporting noise. As the use of EMA and other intensive assessment methods increases, it would be helpful to see more detailed reporting of processes for item construction, participant instructions, and pilot testing.

An advantage of EMA is its ability to assess experiences as they occur, using item wording that focuses on the present moment. In this study, we are intentionally using a different frame of reference (ie, “since you woke up/in the past three hours,” to capture time since the previous prompt) to assess social interactions, social comparisons, and mood state. This decision was based on the low likelihood of capturing social interactions and social comparisons in the moments that they occurred, as participants were not expected to interrupt social activities to complete the surveys. This approach also allows survey responses to capture events in the very recent past, rather than missing them if they are not currently happening. Similarly, we expected mood state to fluctuate throughout the day, and for mood states that predominated over 3-hour spans to be more predictive of PA than immediate emotions (which might be fleeting). For this reason, mood state was assessed as a summary of the past three hours, rather than as a reflection of mood state at the immediate moment of survey completion. Although decisions about the time frames for these reports were based on existing research and our specific research questions, it is important to acknowledge the associated limitations. Primarily, recall bias and forgetting are inherent in any retrospective report, and it will be possible that reports be skewed toward salient experiences or underestimate their true frequency in the natural environment [129,144]. However, to remain consistent with existing EMA studies of body image [130,145,146], we focus our assessment of body satisfaction on participants’ immediate experiences. Consequently, interpretation of findings will require attention to this difference between reporting time frames.

An early consideration involved the method of distribution of EMA surveys to participants’ personal smartphones. Although professional services exist to manage survey distribution (and were considered for Project WHADE), payment regulations presented barriers to hiring outside assistance. Consequently, as in our pilot work, survey scheduling and distribution were managed by study staff, via Qualtrics. A strength of this method is that it offers researchers a great deal of control over and insight into the distribution process. However, this method is time-consuming for study staff and may be prone to error, and it requires additional time for rechecking to limit mistakes. The effectiveness of this method also depends on the cell phone carrier and network availability. For example, carriers periodically experience temporary outages during which text messages are not received. As individuals with certain coverage are not able to receive text messages from Qualtrics, a subset of participants are set up to receive EMA surveys via email and are asked to ensure that their smartphone email notifications mirror those of their text messages. To address this difference between participants, the method of survey delivery will be examined as a moderator of survey completion and responses and will be included as a covariate if it shows significant associations with either of these variables.

In addition, it is common for each survey to appear in a unique text message (rather than as a threaded conversation), and a small subset of participants has encountered confusion and completed the wrong survey when prompted. To ensure that the final dataset is accurate, survey numbers are coded manually by study staff based on their completion time stamps, and duplicates are deleted.

### Significance of Project WHADE

Despite these challenges, this study should provide useful insights into midlife women’s PA. Project WHADE is one of a limited number of studies that use EMA to identify within-person variability in and predictors of PA [114]. Unlike traditional (between-person) methods, this approach acknowledges that PA engagement varies within persons from day to day and throughout the day and that the pattern of PA variation may differ between people. As such, this study is powered to detect moderate effects of both within-person relations between proposed predictors and PA and between-person moderators of these relations. To our knowledge, this is the first study to use an EMA approach to understanding influences on PA among midlife women with elevated risk for CVD. This is a population for whom increased PA would have particularly meaningful health benefits, and despite considerable effort to date, few effective PA interventions exist for this group.

An additional advantage of EMA (and similar designs) is that it can be useful for identifying both group- and individual-level patterns. For example, it is possible that some midlife women are more likely to engage in PA after making an upward comparison, whereas others are more likely to engage in a downward comparison. As such, if a social comparison was included as a behavior change technique to be harnessed in future interventions for this population, it is possible that providing only 1 type of comparison opportunity (eg, exposing all participants to upward targets) would work well for some participants and not others. The use of EMA to identify these differences, which represent individual differences in within-person relations, could inform the tailoring of the content provided by future interventions to particular subgroups and individuals [54]. Thus, an examination of PA at the within-person level could improve our basic understanding of the relations between day-to-day experiences and cardioprotective behavior in an at-risk group and potentially help to identify and clarify appropriate targets for tailored interventions for this population.

Together, these features of Project WHADE suggest that it is poised to address gaps in the current understanding of within-person processes associated with PA variability. We hope that a greater understanding of the within-person predictors of change in PA will provide information useful to the design of interventions, including novel just-in-time approaches that can respond to participants in real time [54]. Although Project WHADE will provide this information for a particular subset of adults (ie, midlife women with cardiovascular risk markers), the overall approach may serve as a model for future investigations of PA determinants and their potential for translation to intervention.

## Appendix

Multimedia Appendix 1 Peer review report.

## Abbreviations

CVD: cardiovascular disease
EMA: ecological momentary assessment
PA: physical activity
WHADE: Women’s Health And Daily Experiences
