# Supplementary material for: Rationale and Design of the Women’s Health And Daily Experiences Project: Protocol for an Ecological Momentary Assessment Study to Identify Real-Time Predictors of Midlife Women’s Physical Activity
Source: JMIR Res Protoc. 2020 Oct 15;9(10):e19044. doi: 10.2196/19044 (PMC7596655; doi:10.2196/19044)
Supplement: Multimedia Appendix 1 [file resprot_v9i10e19044_app1.pdf]

SUMMARY STATEMENT
(Privileged Communication)

PROGRAM CONTACT:
Josephine Boyington
301-594-2542
boyingtonje@mail.nih.gov

Release Date: **07/24/2017**
Revised Date:

Application Number: **1 K23 HL136657-01A1**

Principal Investigator

ARIGO, DANIELLE R

Applicant Organization: UNIVERSITY OF SCRANTON

Review Group:	**MPOR (OA)**<br>**NHLBI Mentored Patient-Oriented Research Review Committee**

Meeting Date:	**06/22/2017**	***RFA/PA:***	**PA16-198**
Council:	**OCT 2017**	***PCC:***	**HHCP N**
Requested Start:	**12/01/2017**	***Dual PCC:***	**RAJ DUAL**
		Dual IC(s):	**DK**

Project Title:	**Identifying and Targeting Unique Physical Activity Determinants for Midlife Women**
SRG Action:	[redacted]
Next Steps:	**Visit https://grants.nih.gov/grants/next_steps.htm**
**Human Subjects:**	**30-Human subjects involved - Certified, no SRG concerns**
**Animal Subjects:**	**10-No live vertebrate animals involved for competing appl.**
**Gender:**	**2A-Only women, scientifically acceptable**
**Minority:**	**1A-Minorities and non-minorities, scientifically acceptable**
**Children:**	**3A-No children included, scientifically acceptable**
	**Clinical Research - not NIH-defined Phase III Trial**

[redacted]

ADMINISTRATIVE BUDGET NOTE: The budget shown is the requested budget and has not been adjusted to reflect any recommendations made by reviewers. If an award is planned, the costs will be calculated by Institute grants management staff based on the recommendations outlined below in the COMMITTEE BUDGET RECOMMENDATIONS section.

1 K23 HL136657-01A1 ARIGO, DANIELLE

NHLBI "K" series resubmission/amended application due dates are Mar. 12, July 12, and Nov. 12.

As a reminder, effective for due dates on or after January 25, 2016, most Career Development Awards (with the exception of K02, K05, and K24) are required to address Rigor and Transparency as part of the research plan. Please refer to NOT-OD-16-012 for implementation instructions.

A change in the NIH definition of a child is outlined in new notice on Inclusion of Children in Clinical Research: Change in NIH Definition.

NIH has a new policy on Appendix Materials, effective for due dates on or after January 25, 2017, which now significantly limits the types of materials allowed to be submitted as application appendices.

RESUME AND SUMMARY OF DISCUSSION: This is a resubmission of a K23 from Danielle Arigo, a clinical psychologist, who has proposed to study the psychosocial barriers to physical activity for mid-life women and to develop a mobile health app to encourage activity. The Candidate continues to be a major strength of the current submission. While she is located at a university underrepresented in federal research funding, she has been successful in applying for intramural research funding and has recruited mentors from outside her university for the proposed studies (which was a point of discussion, but in the end, was thought to be more of a demonstration of her commitment to doing research rather than a potential weakness). Her Career Development Plan is well developed and she has eliminated training for programming/app development, and states that she will now collaborate with experts from her university. However, the Candidate did not provide assurances that a specific individual will collaborate with her – there is no letter of support provided to attest to this. This uncertainty and lack of specific details regarding app development (and funding) and, importantly, app maintenance/upgrading, was viewed as a serious weakness in the application. While the Candidate has been very responsive to many of the previous concerns, poor planning for the App development significantly decreased reviewer confidence for success, and in this project as a vehicle to help her reach research independence.

DESCRIPTION (provided by applicant): Cardiovascular risk and mortality is uniquely elevated among midlife women due to factors such as menopause and gender differences in cardiovascular event symptom presentation. Physical activity (PA) is critical to reducing risk in midlife women; however, this group confronts distinctive psychosocial barriers to PA that rarely are addressed by existing interventions. Current evidence and the candidate's pilot data indicate that these barriers are negative mood, body satisfaction, and social comparison (i.e., evaluating one's PA relative to that of peers). Mobile health tools such as smartphone applications have the ability to intervene on these barriers in real time, but developing effective tools requires a sophisticated understanding of (1) when PA occurs and determinants of PA for particular individuals in their natural environments, and (2) the ability to optimize this information for mobile delivery. The proposed K23 training program will address these needs by focusing on the following training goals: ambulatory assessment of PA and its psychosocial determinants, advanced statistical methods, individual tailoring of PA programs (with an emphasis on mobile intervention development), women's health, and cardiovascular physiology. Training will include apprenticeships and tutorials with experts in each of these topics, as well as coursework in intensive ambulatory assessment, mobile application design, and physiology.

To support this plan, the proposed research will investigate PA and its determinants among midlife female primary care patients with cardiovascular risk markers. Aim 1 of this research is to examine relations between real-time psychosocial experiences (i.e., mood, body satisfaction, social comparison)

and PA among at-risk midlife women (n=100, age 40-60; 27>BMI<50 with one additional cardiovascular risk marker). This will allow for further optimization of a mobile health tool tailored for midlife women. Aim 2 of this research is to refine and pilot test a novel mobile PA application tailored for at-risk midlife women (n=30, age 40-60; 27>BMI<50 with one additional CVD risk marker). Achieving these project aims will produce multiple peer-reviewed manuscripts and a strong R01 proposal focused on testing a well-informed, tailored mobile intervention for midlife women at risk for developing CVD. This research builds on the candidate's training to date and introduces an innovative way to understand and promote PA in midlife women. The goals of this K23 program are directly responsive to NHLBI's strategic plan, as they will allow for improved primary prevention through increased understanding of determinants of disease risk, and the proposed program will contribute to improving interventions tailored to patient needs and barriers. The excellent training, protected time, and project resources of the proposed K23 program will prepare the candidate to become a leading patient-oriented researcher with expertise in PA promotion for cardiovascular risk reduction among midlife women.

PUBLIC HEALTH RELEVANCE; Midlife women are at unique risk for cardiovascular disease, in part due to physical inactivity, and innovative approaches are needed to promote activity in this group. Existing mobile health tools do not target midlife women's distinct physical activity determinants in real time, and rarely are developed or evaluated with input from behavioral scientists. Training in real-time physical activity assessment and optimization of mobile interventions for this group will prepare the candidate to be a leading patient-oriented researcher in physical activity promotion for cardiovascular risk reduction among midlife women.

CRITIQUE 1;

[redacted]

Overall Impact:
This is a revised application from an outstanding, very well published, candidate, a PhD Assistant Professor of Psychology at the U of Scranton, who is interested in developing an independent career optimizing individually tailored interventions to promote cardioprotective behaviors in midlife women. Her K23 proposal focuses on identifying determinants of impediments to physical activity in mid-life women and incorporating them into mobile health apps. She is addressing an important public health problem. She is partially responsive to the previous critique in that she now includes a conceptual model, the Aims are no longer interdependent, and she has increased the N studied significantly. Her response to the concern expressed by the Reviewers of her A0 application that her training plan did not include adequate training in app development was to remove that aspect of her Career Development Plan and to instead state that she will work with software engineers, but there is no budget item for this, no letter of commitment and no description of the specific expertise or personnel who could provide this service.

1. Candidate:
Strengths

- Assistant Professor of Psychology at University of Scranton since 2014, on a tenure track.
- Has published 15 first-author original research papers.
- Has a strong history of applying for grants (including an R21) and has received several small pilot grants.

- The candidate's success despite a lack of onsite mentors is impressive and speaks to her potential to become an independent clinical researcher, as well as her intellect, initiative, energy and drive.

Weaknesses
- None

2. Career Development Plan/Career Goals & Objectives/Plan to Provide Mentoring:
Strengths
- Candidate has developed a Career Development Plan to train her in the following areas:
- (1) momentary assessment methods and corresponding statistical approaches, (2) ambulatory physical activity assessment methods (emphasizing work with raw accelerometer data and parameters for midlife women), (3) individually tailored intervention design with an emphasis on translation to mHealth tools, and (4) cardiovascular exercise physiology.
- For most of the training areas identified above, the Career Development Plan includes tutorials and didactics.
- Candidate currently has a required 3-course per semester teaching load. The K23 support would buy back some of that time, allowing her to engage in training and research activities more fully.

Weaknesses
- None

3. Research Plan:
Strengths
- The candidate is addressing an important public health problem: the contribution of physical inactivity to cardiovascular risk and the determinants of this physical inactivity. In the first Aim, she will investigate the determinants of physical activity in women during midlife and in the second she will refine and pilot test a mobile application in overweight/obese midlife women with one additional CV risk factor.
- Aim 2 is not dependent on Aim 1 because she already has pilot data upon which to base the Aim 2 mobile app work.
- Has considered potential pitfalls and limitations.
- Has thought about how the K23 project could generate preliminary data for an R01 application (in which she would propose a large-scale efficacy trial of her app).
- Letter of collaboration from Prime Med Medical Group should help with study subject recruitment.

Weaknesses
- Reviewers of the A0 version of the K23 noted as a weakness that although a main goal of the candidate was to develop a mobile app, she had no training or plan for training in this area. Her response in this A1 application is that she will not be trained in this area but instead will work with software engineer at the University of Scranton, which she states has the expertise and is committed to provide these services. Yet there is no budget item for this, no specific collaborators identified with expertise in this area and no letter of collaboration between a specific software engineer. There is no description of the specific expertise or personnel who could provide this service. The sentence in the Institutional Commitment letter stating that she has "access to cutting-edge software development resources through our Computing Sciences Department and Software Engineering M.S. program, which is committed to supporting this work (e.g. offering consulting services as needed) is helpful but not entirely convincing. Identification of a collaborator who attested to his/her commitment to this project would have been a stronger response and would have gone a long way toward making it clear that the proposed project is feasible.

- The candidate states that she will refine instead of creating an app, but there are no details regarding what existing app would form the basis of her new intervention. This also speaks to feasibility.
- The candidate's plan for her R01 application relies on success of her mobile app aim.

4. Mentor(s), Co-Mentor(s), Consultant(s), Collaborator(s):
Strengths
- Primary mentor, Dr. Smyth, is a well-published, highly respected professor, who has an NIH grant and is a co-investigator on several others, and has published widely with the candidate over many years (see below).
- The candidate has designated a specific and frequent meeting schedule with her mentors.

Weaknesses
- Lack of mentorship available at her home institution, which is largely an undergraduate institution, is a limitation of this proposal. To her credit, however, she has assembled a team of mentors from institutions that she states are less than 3 hours from U of Scranton, including her scientific mentor, Joshua Smyth, who is at the Pennsylvania State University. Although arrangements in which the primary mentor is distant from the mentee are almost always ineffective, in this case, there is a proven track record of co-authorship from 2008-2016, which mitigates some of these concerns.

5. Environment and Institutional Commitment to the Candidate:
Strengths
- Salaried, tenure-track appointment, with a guaranteed 3-credit course release each year to conduct research.
- Had a start-up package.
- Candidate has been awarded 3 internal Faculty Development grants.
- Description of Environment is now more detailed and more reassuring with regard to resources available to support the candidate's proposed research.

Weaknesses
- Teaching load is heavy, and relief sufficient to provide at least 75% protected time for research is contingent on the awarding of the K23 grant.

Inclusion of Women, Minorities and Children:
- Justification for exclusion of men has been enhanced and is now compelling.

Resubmission:
- Responsive in some ways but not in others, as described above.

CRITIQUE 2:

[redacted]

Overall Impact:
The applicant was highly responsive to critiques from the last review. Specifically, changes and clarifications to the research plan have considerably strengthened the overall proposal from this excellent applicant.

1. Candidate:
Strengths

- As noted regarding the previous submission, the applicant has excellent potential for developing as an independent and productive researcher focusing on patient-oriented research. The candidate has outlined a logical progression of research, based on previous pilot work, that indicates a commitment to becoming an independent investigator focusing on patient-oriented research. She has done patient-oriented research in several settings. Letters of reference are very strong and indicate the high potential of the candidate for becoming an independent investigator.

Weaknesses

- None noted.

2. Career Development Plan/Career Goals & Objectives/Plan to Provide Mentoring:
Strengths

- It was previously noted as a minor weakness that mentors are scattered among three institutions. On reflection, this is viewed as a strength: mentors demonstrate an exceptionally strong investment in the applicant, and the applicant demonstrates an exceptionally strong commitment to patient-oriented research.
- Weaknesses noted in the previous submission have been adequately addressed. Specifically, the new emphasis on training in the translation of behavioral science principles to mobile health tools is a strength.

Weaknesses

- None noted.

3. Research Plan:
Strengths

- Overall the research plan articulates a clear question that the candidate is uniquely situated to answer and has been working toward with pilot work. It is a logical next step in a progression that will launch the candidate toward her career objectives.
- Most weaknesses noted in the previous submission have been adequately addressed, and the plan is generally strong.

Weaknesses

- In the pilot intervention, some prompts will require users to input psychosocial factors, and others will provide intervention content selected at random. I'm curious why the applicant didn't consider linking the two, such that intervention messages would be selected based on inputted psychosocial factors. This may increase programming complexity but also takes full advantage of the mobile health platform. The applicant does indicate that specifications are subject to modification based on knowledge developed via career development activities, so this or other options may be considered as the project unfolds.
- It has been clarified that the applicant will achieve the software development via collaboration with the computing resources available at the University of Scranton. However, while within the same university, it hasn't been demonstrated that these resources can be feasibly accessed, for example by a Letter of Support from this department. Since there are no budgetary resources for software development, this type of arrangement can often depend heavily on faculty and student interest which is not conclusively demonstrated. There is a statement in the letter from the Dean of the College of Arts and Sciences indicating that the Computing Sciences Department is committed to supporting this work, however, so this is considered a relatively minor concern.

4. Mentor(s), Co-Mentor(s), Consultant(s), Collaborator(s):
Strengths

- The candidate has assembled an excellent, multidisciplinary team of mentors with proven records of successful research and mentoring.
- Mentors have articulated strong support for the candidate and adequate plans for monitoring and evaluating the awardee's progress toward independence.

Weaknesses
- None noted.

5. Environment and Institutional Commitment to the Candidate:
Strengths
- The infrastructure at the candidate's home institution is clearly articulated in the resubmission.
- The institution has documented support for the candidate's effort to the research at 77%.

Weaknesses
- As noted with the research plan, there is some lingering concern about adequacy of the software development resources.

Inclusion of Women, Minorities and Children:
- There was concern in the last review regarding justification for the exclusion of men. The applicant has made the justification for a women-only study exceedingly explicit in the resubmission.

Resubmission:
- The applicant has been highly responsive to the critiques from the last review.

Budget and Period of Support: Recommend as Requested
- It would be optimal to include at least some funds for programming (for example, to support a student internship under faculty supervision) to help ensure feasibility of creating the mHealth platform.

CRITIQUE 3:

[redacted]

Overall Impact:
This is a resubmitted application from a candidate who wishes to establish an independent research career in physical activity interventions among mid-life women, with special emphasis on mHealth applications. Overall, applicant was somewhat responsive to prior critiques, but the translation of information on psychosocial factors related to physical activity into mobile application development is still not well described (as far as who will assist in this key step). In addition, while mentors are well qualified overall, several are remote which is not optimal. There were also some weaknesses in the research plan that decreased enthusiasm. The overall impact on a successful application to effectively increase activity in this population would be very high.

1. Candidate:
Strengths
- PhD in Clinical Psychology from Syracuse University
- Postdoctoral fellowship in Clinical Health Psychology at Drexel University
- Currently Assistant Prof of Psychology at Scranton University.

- Has a number of pending R01 applications that she may receive support from.

Weaknesses

- Has a number of first author publications, though not many that are directly relevant to the proposed research.

2. Career Development Plan/Career Goals & Objectives/Plan to Provide Mentoring:

Strengths

- Some didactic coursework in physiology.
- Tutorials and regular meetings with mentors.
- NIH sponsored mHealth training.
- R01 development in Year 4.

Weaknesses

- There is still some concern that translation of the knowledge to a mobile app will require collaboration with expert software engineers, and none are named. It seems it may be important to identify individuals with expertise in developing apps targeting behavior change.
- Most mentors are remote, although plans are in place to meet weekly via phone or Skype.

3. Research Plan:

Strengths

- Aim 1 will explore real-time associations of psychosocial experience with physical activity (based on questionnaire and accelerometry).
- Aim 2 is to develop and pilot test an application to improve physical activity by addressing psychosocial experiences.
- Could have high impact if successful.

Weaknesses

- Sleep and fatigue are not addressed as factors that could impact mood as well as physical activity levels, particularly in overweight and obese middle-aged women with risk factors for CVD.
- Inclusion/exclusion criteria not addressed in research plan, although mentioned in human subjects.
- Little mention of possible covariates and factors other than psychosocial experience that could impact PA (e.g., what if they have caregiving responsibilities, or live in an area where it is not safe to be outdoors?)

4. Mentor(s), Co-Mentor(s), Consultant(s), Collaborator(s):

Strengths

- Overall team has appropriate expertise and well-funded.

Weaknesses

- No detailed accounting of mentees and numbers with K awards, academic appointments, etc. although described in general.
- Most mentors are remote and phone meetings will be necessary.

5. Environment and Institutional Commitment to the Candidate:

Strengths

- Environment seems adequate to carry out the research for the most part

Weaknesses

- Unclear where the application development will take place, and expertise of the engineers who will assist with this.

Inclusion of Women, Minorities and Children:

- Focus of the proposal is increasing activity in mid-life women, therefore men are excluded as are children.

THE FOLLOWING SECTIONS WERE PREPARED BY THE SCIENTIFIC REVIEW OFFICER TO SUMMARIZE THE OUTCOME OF DISCUSSIONS OF THE REVIEW COMMITTEE, OR REVIEWERS' WRITTEN CRITIQUES, ON THE FOLLOWING ISSUES:

PROTECTION OF HUMAN SUBJECTS (RESUME): ACCEPTABLE

INCLUSION OF WOMEN PLAN (RESUME): ACCEPTABLE; women only, scientifically justified.

INCLUSION OF MINORITIES PLAN (RESUME): ACCEPTABLE

INCLUSION OF CHILDREN PLAN (RESUME): ACCEPTABLE; no children involved, scientifically justified.

TRAINING IN THE RESPONSIBLE CONDUCT OF RESEARCH: ACCEPTABLE

RESOURCE SHARING PLANS: NOT APPLICABLE (NO RELEVANT RESOURCES)

AUTHENTICATION OF KEY BIOLOGICAL AND/OR CHEMICAL RESOURCES: NOT APPLICABLE (NO RELEVANT RESOURCES)

COMMITTEE BUDGET RECOMMENDATIONS: RECOMMENDED AS REQUESTED

Footnotes for 1 K23 HL136657-01A1; PI Name: Arigo, Danielle R

NIH has modified its policy regarding the receipt of resubmissions (amended applications). See Guide Notice NOT-OD-14-074 at http://grants.nih.gov/grants/guide/notice-files/NOT-OD-14-074.html. The impact/priority score is calculated after discussion of an application by averaging the overall scores (1-9) given by all voting reviewers on the committee and multiplying by 10. The criterion scores are submitted prior to the meeting by the individual reviewers assigned to an application, and are not discussed specifically at the review meeting or calculated into the overall impact score. Some applications also receive a percentile ranking. For details on the review process, see http://grants.nih.gov/grants/peer_review_process.htm#scoring.
